# Editorial for the Special Issue on SiC Based Miniaturized Devices, 3rd Edition

**DOI:** 10.3390/mi17060638

**Published:** 2026-05-22

**Authors:** Stephen E. Saddow, Brenda L. VanMil, Benoit Hamelin

**Affiliations:** 1 Department of Electrical Engineering, University of South Florida, 4202 East Fowler Avenue, ENB118, Tampa, FL 33620, USA; 2 DEVCOM Army Research Laboratory, 2800 Powder Mill Rd., Adelphi, MD 20783, USA; brenda.l.vanmil.civ@army.mil; 3 EngeniusMicro, Huntsville, AL 35801, USA; benoit.hamelin@engeniusmicro.com

Silicon Carbide: Enabling Next-Generation Power Electronics and High-Performance Computing.

The past two years have shown that Silicon Carbide is no longer an emerging technology: it is a fully ascendant force shaping the future of electrification, high-power computing, and advanced manufacturing. With global megafabs coming online, substrate and defect-control breakthroughs accelerating, and SiC penetrating deeper into automotive, industrial, and energy infrastructures, the material has reached a defining moment. For the first time, the world is preparing to manufacture it at the scale necessary to power the next generation of energy and computing systems.

Major Accomplishments (2024–2026)

- Global transition to 200 mm SiC wafers, led by major multibillion-dollar fab expansions in the U.S., Europe, and Asia [1].
- Unprecedented investment momentum, driven by national semiconductor initiatives and accelerating electrification [2,3,4].
- Market growth trajectory exceeding $10 billion by 2030, underpinned by EVs, renewable energy, and high-density data centers [5].
- Breakthrough substrate technologies, including SmartSiC engineered substrates and advanced composite wafer designs that reduce stress, cost, and defectivity [6,7].
- Strengthened global supply chain, with new crystal-growth facilities, vertically integrated EV module development, and high-volume SiC power-module packaging capabilities [8].
- Expansion beyond power electronics, with new activity in quantum sensing, advanced materials engineering, eco-friendly SiC synthesis, and multifunctional composite applications [9,10,11,12,13,14].

This volume, drawn from the Special Issue “SiC Based Miniaturized Devices, 3rd Edition” of *Micromachines*, and edited by Prof. Dr. Stephen E. Saddow, Dr. Brenda L. VanMil, and Dr. Benoit Hamelin, offers a comprehensive snapshot of the rapid progress and expanding scope of Silicon Carbide (SiC) technologies. The contributions assembled here reflect a vibrant and multidisciplinary research landscape, encompassing material growth, device physics, advanced modeling, and device reliability in extreme environments. Collectively, these works reaffirm SiC’s position as a critical enabling material for next-generation miniaturized devices and high-performance systems.

The opening paper, “Model of Quality Factor for (111) 3C-SiC Double-Clamped Beams” (contribution 1), provides a rigorous investigation into the performance limits of SiC-based MEMS resonators. By combining experimental datasets with both analytical and numerical models, the authors elucidate the role of crystallographic defects at the 3C-SiC/Si interface in determining the quality factor. The identification of a minimum thickness threshold for optimal device performance offers a clear and actionable design guideline, bridging material science and MEMS engineering.

Expanding into high-voltage device innovation, “A 3.3 kV SiC Semi-Superjunction MOSFET with Trench Sidewall Implantations” (contribution 2) introduces a practical semi-superjunction architecture that balances enhanced electrical performance with fabrication feasibility. Through detailed TCAD simulations, the authors demonstrate meaningful improvements in breakdown voltage and on-state resistance, particularly in trench-based configurations. This contribution highlights the importance of manufacturable design strategies in advancing SiC power electronics.

The third paper, “Analyzing the Impact of Gate Oxide Screening on Interface Trap Density in SiC Power MOSFETs Using a Novel Temperature-Triggered Method” (contribution 3), addresses a long-standing challenge in device characterization. The proposed temperature-triggered threshold voltage shift (T3VS) method offers a streamlined approach to extracting interface trap density without requiring extensive prior knowledge of device structure. The findings emphasize the superior interface quality of trench devices while also cautioning against aggressive screening techniques that may inadvertently introduce defects, thereby underscoring the delicate balance between performance optimization and reliability.

In “A High-Density 4H-SiC MOSFET Based on a Buried Field Limiting Ring with Low Qgd and Ron” (contribution 4), the authors present a novel structural design that significantly enhances device efficiency. By incorporating a grounded P+ shield within a split trench architecture, the proposed MOSFET effectively mitigates electric field concentrations at the gate oxide. The resulting improvements in switching charge and figure of merit demonstrate how thoughtful structural innovation can overcome traditional trade-offs in device design.

Complementing these developments, “Simulation Study on 6.5 kV SiC Trench Gate p-Channel Superjunction Insulated Gate Bipolar Transistor” (contribution 5) explores the application of superjunction concepts to p-channel IGBTs. The work reveals substantial gains in forward conduction and switching behavior, while also analyzing temperature-dependent effects and performance trade-offs. These insights are particularly valuable for the development of ultra-high-voltage devices intended for demanding operational environments.

The importance of accurate modeling is further emphasized in “Numerical Simulations of 3C-SiC High-Sensitivity Strain Meters” (contribution 6), where the authors introduce anisotropic damping formulations to better capture energy dissipation in dynamic systems. By moving beyond conventional isotropic assumptions, this work enhances the predictive power of simulations and improves alignment with experimental observations, particularly in complex and directionally dependent materials.

Reliability under extreme conditions is a defining strength of SiC technologies, as demonstrated in “SPICE Model for SiC Bipolar Transistor and TTL Inverter Degradation Due to Gamma Radiation” (contribution 7). This study presents a calibrated SPICE model that connects radiation-induced material degradation to circuit-level performance. The demonstrated resilience of SiC devices under high radiation exposure, along with predictive lifetime modeling, underscores their suitability for space, nuclear, and other radiation-intensive applications.

Finally, “Optimization and Simulation on Gas Flow and Temperature Fields on the Homoepitaxial Growth of N-Doped 4H-SiC Wafers” (contribution 8) addresses the foundational challenge of material uniformity. By systematically analyzing chemical vapor deposition parameters and their influence on nitrogen doping profiles, the authors identify the conditions necessary for achieving homogeneous epitaxial layers. This work provides essential guidance for scaling SiC wafer production while maintaining the material quality required for advanced device applications.

Across all contributions, a unifying theme emerges: the critical interplay between material quality, device architecture, and modeling accuracy. Progress in SiC technology is shown to depend not on isolated advances, but on the integration of insights across these domains. From the control of defects at the atomic scale to the optimization of device performance and reliability at the system level, each paper contributes to a deeper and more cohesive understanding of the field. As SiC continues to transition from a specialized material to a mainstream platform for miniaturized and high-power devices, the importance of such integrated research efforts cannot be overstated. Future developments will likely build upon the methodologies and innovations presented here, further enhancing performance, scalability, and robustness.

In conclusion, this Special Issue captures both the current state and the future trajectory of SiC-based miniaturized devices. The editors were delighted to assemble a collection that not only advances the field but also inspires continued exploration and collaboration across disciplines. The work presented in this volume will undoubtedly serve as a valuable reference for researchers and engineers striving to push the boundaries of what SiC technology can achieve.
